# Association between serum carotenoids and bacterial vaginosis infection among American women

**DOI:** 10.1186/s12879-023-08908-3

**Published:** 2024-01-02

**Authors:** Ming-Zhi Tan, Yu-Xue Feng, De-Yao Hong, Xu-Guang Guo

**Affiliations:** 1 Department of Clinical Laboratory Medicine, Guangdong Provincial Key Laboratory of Major Obstetric Diseases; Guangdong Provincial Clinical Research Center for Obstetrics and Gynecology, The Third Affiliated Hospital of Guangzhou Medical University, Guangzhou, 510150 China; 2 Department of Clinical Medicine, The Third Clinical School of Guangzhou Medical University, Guangzhou, 511436 China; 3 Guangzhou Key Laboratory for Clinical Rapid Diagnosis and Early Warning of Infectious Diseases, King Med School of Laboratory Medicine, Guangzhou Medical University, Guangzhou, 510000 China; 4 Department of Clinical Medicine, The First Clinical School of Guangzhou Medical University, Guangzhou, 511436 China

**Keywords:** Bacterial vaginosis, Serum carotenoids, NHANES, Health, Immunomodulatory, Antioxidant

## Abstract

**Background:**

Bacterial vaginosis (BV) is a widely occurring vaginal inflammation in women of childbearing age caused by dysbiosis of the vaginal flora. Few studies have investigated the effect of serum carotenoids on the development and pathogenesis of BV. This study thus aimed to explore the correlation between serum carotenoids and BV in American women.

**Method:**

The analysis included 1252 participants with BV from the National Health and Nutrition Examination Survey (NHANES) between 2001 and 2004. Multiple logistic regression was conducted to explore the correlation between BV and serum carotenoids, while smooth curve fitting was utilized to examine potential nonlinear correlations. Furthermore, stratified subgroup analyses and sensitivity analyses were conducted. ORs reflected the correlation between BV and serum carotenoids.

**Result:**

Results of multiple logistic regression indicated that total serum carotenoids and BV had an inverse correlation. In the fully adjusted model II, the quartile with the highest levels of α-carotene and β-cryptoxanthin had a substantially lower incidence of BV. Smooth curve fitting revealed a significant negative linear correlation between serum carotenoids and the incidence of BV. The negative correlation between serum carotenoids and BV was relatively stable in stratified analyses. Moreover, in sensitivity analyses, the association between serum carotenoids and BV persisted, and β-carotene became significantly negatively correlated with BV.

**Conclusion:**

This study found an inverse correlation between serum carotenoids and the prevalence of BV.

**Supplementary Information:**

The online version contains supplementary material available at 10.1186/s12879-023-08908-3.

## Introduction

Bacterial vaginosis (BV) is a mixed infection caused by an imbalance in the normal flora of the vagina, resulting in a syndrome of itching and burning of the vulva, increased and thin vaginal discharge, and fishy-smelling leukorrhea [1]. The prevalence of BV varies widely among women worldwide, ranging from 4 to 75%, with an approximate rate of 30% in the United States [2, 3]. The treatment for BV is often effective, but it is prone to relapse after discontinuation of the medication [4, 5]. In addition to causing physical discomfort in women, BV may raise their risk of contracting HIV, preterm labor, uterine fibroids, endometriosis, surgical adhesions, and other gynecologic disorders [6–8].

Carotenoids are one of the micronutrients in the human diet with anti-inflammatory, antioxidant, and immunomodulatory properties. Research has linked them to conditions such as depression [9], respiratory disease [10], and other chronic diseases [11–14]. Carotenoids are known to mitigate oxidative stress by directly quenching free radicals, reducing damage caused by reactive oxygen species, and preventing lipid peroxidation [15]. They are also involved in cellular communication and maintenance of epithelial cell integrity [16]. Studies have suggested that women with cervical intraepithelial neoplasia and cervical cancer exhibit notably lower levels of β-carotene in cervicovaginal cells and plasma [17]. Furthermore, carotenoids have also demonstrated the capacity to enhance immune functions, including the stimulation of lymphocyte proliferation, the release of cytokines, and the cytotoxic activity of natural killer cells [18].

Although the etiology of BV is multifaceted and not yet well researched, there is evidence to suggest that the progression of BV is accompanied by the accumulation of reactive oxygen species and alterations in immunometabolism [19, 20]. Theoretically, carotenoids could potentially impact the development of BV by mitigating oxidative stress, preserving the integrity of the vaginal epithelium, and modulating immune function [21, 22]. However, there have been a limited number of relevant studies investigating the effect between serum carotenoids and BV. Hence, the objective of this study was to explore the association between different serum carotenoids and BV by utilizing NHANES data collected from 2001 to 2004.

## Method

### Study population

All data are available from the NHANES database, a series of research designed to evaluate the health status of the citizens and ambulatory populations in America [23]. Data from two survey cycles, 2001–2002 and 2003–2004, were adopted in this study.

**Fig. 1:**
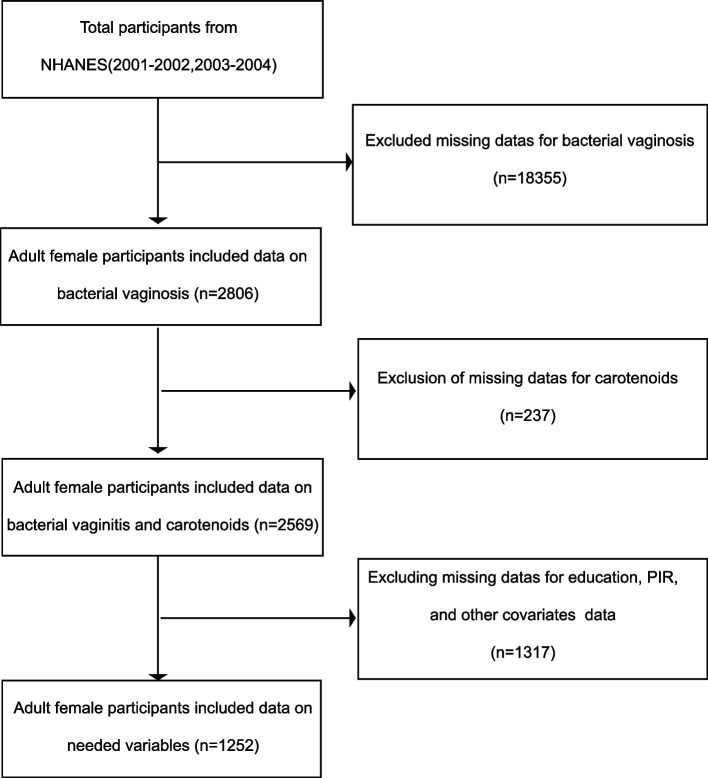
Research flowchart

The participant selection process is illustrated in Fig. 1, involving 21,161 participants over the two survey cycles. Firstly, participants with BV were included, with a total of 2806 study samples. All participants were adult females between the ages of 18–49. Next, we excluded participants with missing carotenoid data (*n* = 237). Finally, participants with missing data on education, PIR, and other covariates were excluded (*n* = 1317). The analysis comprised a total of 1252 eligible participants.

### Diagnostic for BV

The process of BV diagnosis can be known through NHANES documentation [24, 25]. In brief, participants collected vaginal swabs at a mobile examination center after signing a written informed consent. NHANES staff coated the swabs on pH paper and then transferred the swabs onto glass slides. Subsequently, the slides underwent Gram staining and were assessed in a central laboratory using the Nugent criteria. The Nugent Score, which evaluates the vaginal microecology by quantifying the presence of stray bacteria, was utilized. BV was considered present when the Nugent score fell within the range of 7 to 10, while scores from 0 to 6 signified the absence of BV [26]. Women who did not have Nugent scoring system results were excluded. Nugent score data are available in the NHANES database for the 18–49 age group, but data are not publicly available for those under 18.

### Assessment of carotenoids

The NHANES documentation contains measurements of the various serum carotenoids, and high-performance liquid chromatography was applied to assess serum concentrations of α-carotene, β-carotene, β -cryptoxanthin, lycopene, and lutein/zeaxanthin. The six types of carotenoids mentioned above account for over 95% of human serum carotenoids [11]. Quantification is completed by measuring the peak height at 450 nm and then comparing it to the peak height of a standard sample solution. The concentrations of the six prime carotenoids in serum were summed to obtain the total concentration of carotenoids in serum [27, 28].

### Covariates

To decrease the error of the model, we selected the following variables as potential covariates for our study (age, race, education, body mass index (BMI), Poverty Income Ratio (PIR), marital status [29], physical activity [30], C-reactive protein [31], serum vitamin A [32, 33], serum vitamin E [33], serum calcium [32], high cholesterol level [32], sexual intercourse [34], birth control pills [35], smoking status [36], alcohol consumption [37]).

The details of these variables are described as follows.

The study population consisted of five racial categories: Mexican American, Hispanic, non-Hispanic white, non-Hispanic black, and other races. Education status was divided into three categories: below high school education, high school graduation, and above high school education.

Body Mass Index (BMI), calculated as an individual’s weight in kilograms divided by the square of their height in meters (weight (kg) / [height (m)]2), served as the basis for classifying participants into four BMI categories: underweight (BMI less than 18.5), healthy weight (BMI 18.5–24.9), overweight (BMI 25–30), and obesity (BMI greater than 30) [38, 39].

Marital status encompassed various categories, including marriage, widowhood, divorce, separation, never getting married, and cohabitation. Physical activity was categorized into two primary groups: moderate and vigorous, with three supplementary options for each category (yes, no, or unable to do activity).

Participants’ information on sexual intercourse and birth control pill usage was obtained through NHANES questionnaires. Sexual intercourse, as defined in the questionnaire, encompassed vaginal intercourse, oral sex, and anal sex. Participants with total cholesterol values equal to or exceeding 240 mg/dl were categorized as having a high cholesterol level [40]. Respondents were classified as smokers if they had smoked at least 100 cigarettes in their lifetime, and as non-smokers if they had not smoked at least 100 cigarettes throughout their lifetime. Alcohol use status included three groups: nondrinker, moderate alcohol use, and alcoholism, based on the daily drinking criteria established by Ratten et al. [41].

Data of the remaining continuous variables, including C-reactive protein, serum vitamin A, serum vitamin E, and serum calcium, were obtained from the NHANES laboratory dataset.

### Statistical analysis

Multiple logistic regression analyses were conducted for serum carotenoids as a whole and for each of the prime components to explore the associations between serum carotenoids and the incidence of BV. In the analyses, continuous variables that followed a normal distribution were reported using the mean and standard deviation, while those that did not follow a normal distribution were reported using the median. Categorical variables were reported as percentages.

Meanwhile, three models (unadjusted model, model I, and model II) were constructed to enhance the reliability of the findings. The unadjusted model did not incorporate adjustments for any covariates. Model I adjusted for age, race, education status, BMI, marital status, PIR, and physical activity (Moderate and vigorous activity). Based on Model I, Model II added covariates for C-reactive protein, serum vitamin A, serum vitamin E, serum calcium, high cholesterol level, sexual intercourse, birth control pills, smoking status, and alcohol consumption. The quartiles of serum carotenoid levels were determined based on the distribution within the study population, with Q1 ranging from 3.14 to 47.69 μ g/ml, Q2 ranging from 47.77 to 64.39 μ g/ml, Q3 ranging from 64.40 to 85.80 μ g/ml, and Q4 ranging from 85.96 to 331.7 μ g/ml. ORs reflected the correlations between clinical outcomes and exposure.

Smoothed curve fitting plots were drawn to visualize the correlation between serum carotenoids and BV and to explore potential non-linear relationships. Subgroup analyses, stratified by covariates, were conducted to mitigate potential study bias. After excluding missing values for BV and serum carotenoids, missing values in the covariates were filled in by multiple interpolations as a sensitivity analysis.

All of our data were processed and analyzed using EmpowerStats software (www.EmpowerStats.com) and the statistical package R (www.r-project.org). Statistical significance was determined by a two-sided *P* < 0.05.

## Result

### Baseline characteristics of the study population

In Table 1, the study population has the following baseline characteristics. There were significant differences in PIR, various serum levels of carotenoids (excluding lycopene), and vitamin E among the study population based on the presence or absence of BV. Compared with negative BV results, there was a higher proportion of non-Hispanic blacks, participants with less than a high school diploma, obesity, never married, without vigorous activity, birth control pill users, smokers, and alcoholism with BV-positive results. In addition, age, lycopene, C-reactive protein, serum calcium, high cholesterol level, and sexual intercourse were not statistically significant (*P* > 0.05).

**Table 1:** Baseline characteristics of participants with Bacterial vaginosis

Characteristics	Bacterial vaginosis (BV) Overall	Negative (Nugent-BV ≤ 6)	Positive (Nugent-BV ≥ 7)	*P*-value
N	1252	877	375	
Age (years), mean ± SD	33.91 ± 8.53	33.86 ± 8.44	34.03 ± 8.76	0.751
PIR Median (Min-Max)	2.67 (0.00–5.00)	3.09 (0.00–5.00)	1.86 (0.00–5.00)	< 0.001
Serum carotenoids (μ g /ml), Median (Min-Max)	63.50 (3.14–262.46)	66.10 (3.14–262.46)	57.80 (13.60–219.40)	< 0.001
α-Carotene (μ g /ml), Median (Min-Max)	2.60 (0.21–64.40)	3.00 (0.21–64.40)	1.91 (0.21–49.11)	< 0.001
β-Carotene (μ g /ml), Median (Min-Max)	12.69 (0.79–146.30)	13.74 (0.79–146.30)	10.49 (0.86–97.80)	< 0.001
β-cryptoxanthin (μ g /ml), Median (Min-Max)	7.50 (0.14–65.96)	8.00 (0.14–65.96)	6.30 (1.01–54.59)	0.002
Lycopene (μ g /ml), Median (Min-Max)	21.91 (0.68–79.80)	22.10 (0.68–68.70)	21.50 (3.62–79.80)	0.116
Lutein + zeaxanthin (μ g /ml), Median (Min-Max)	13.59 (0.97–67.40)	14.00 (0.97–67.40)	12.70 (3.50–45.16)	< 0.001
C-reactive protein (mg/dL), Median (Min-Max)	0.24 (0.01–16.30)	0.23 (0.01–16.30)	0.27 (0.01–4.94)	0.141
Vitamin A (μ g /ml), Median (Min-Max)	51.26 (11.94–148.52)	51.90 (11.94–129.50)	49.44 (23.65–148.52)	0.055
Vitamin E (μ g /ml), Median (Min-Max)	209.00 (20.00–1494.00)	199.00 (20.00–1383.00)	234.00 (28.00–1494.00)	< 0.001
Calcium (mg/dl), Median (Min-Max)	9.35 (8.10–10.60)	9.30 (8.30–10.60)	9.40 (8.10–10.60)	0.926
Race/ethnicity(%)				< 0.001
Mexican American	251 (20.05%)	175 (19.95%)	76 (20.27%)	
Hispanic	47 (3.75%)	36 (4.10%)	11 (2.93%)	
Non-Hispanic White	678 (54.15%)	529 (60.32%)	149 (39.73%)	
Non-Hispanic Black	227 (18.13%)	105 (11.97%)	122 (32.53%)	
Other Race	49 (3.91%)	32 (3.65%)	17 (4.53%)	
Education(%)				< 0.001
Under high school	225 (17.97%)	127 (14.48%)	98 (26.13%)	
High school	265 (21.17%)	173 (19.73%)	92 (24.53%)	
More than high school	762 (60.86%)	577 (65.79%)	185 (49.33%)	
BMI (%)				< 0.001
Underweight	34 (2.72%)	29 (3.31%)	5 (1.33%)	
Healthy weight	471 (37.62%)	354 (40.36%)	117 (31.20%)	
Overweight	349 (27.88%)	246 (28.05%)	103 (27.47%)	
Obesity	398 (31.79%)	248 (28.28%)	150 (40.00%)	
Marital status (%)				< 0.001
Marriage	668 (53.35%)	511 (58.27%)	157 (41.87%)	
Widowhood	12 (0.96%)	6 (0.68%)	6 (1.60%)	
Divorce	98 (7.83%)	57 (6.50%)	41 (10.93%)	
Separation	52 (4.15%)	29 (3.31%)	23 (6.13%)	
Never getting married	293 (23.40%)	189 (21.55%)	104 (27.73%)	
Cohabitation	129 (10.30%)	85 (9.69%)	44 (11.73%)	
Moderate activity (%)				0.012
Yes	745 (59.50%)	543 (61.92%)	202 (53.87%)	
No	499 (39.86%)	327 (37.29%)	172 (45.87%)	
Unable to do activity	8 (0.64%)	7 (0.80%)	1 (0.27%)	
Vigorous activity (%)				< 0.001
Yes	464 (37.06%)	358 (40.82%)	106 (28.27%)	
No	773 (61.74%)	507 (57.81%)	266 (70.93%)	
Unable to do activity	15 (1.20%)	12 (1.37%)	3 (0.80%)	
High cholesterol level (%)				0.146
Yes	167 (13.34%)	125 (14.25%)	42 (11.20%)	
No	1085 (86.66%)	752 (85.75%)	333 (88.80%)	
Sexual intercourse				0.226
Yes	1226 (97.92%)	856 (97.61%)	370 (98.67%)	
No	26 (2.08%)	21 (2.39%)	5 (1.33%)	
Birth control pills (%)				0.007
Yes	1018 (81.31%)	730 (83.24%)	288 (76.80%)	
No	234 (18.69%)	147 (16.76%)	87 (23.20%)	
Smoking status(%)				< 0.001
Smokers	579 (46.25%)	375 (42.76%)	204 (54.40%)	
Non-smokers	673 (53.75%)	502 (57.24%)	171 (45.60%)	
Alcohol consumption (%)				< 0.001
Nondrinker	449 (35.86%)	346 (39.45%)	103 (27.47%)	
Moderate alcohol use	401 (32.03%)	285 (32.50%)	116 (30.93%)	
Alcoholism	402 (32.11%)	246 (28.05%)	156 (41.60%)	

### The association of total serum carotenoids with BV

**Fig. 2:**
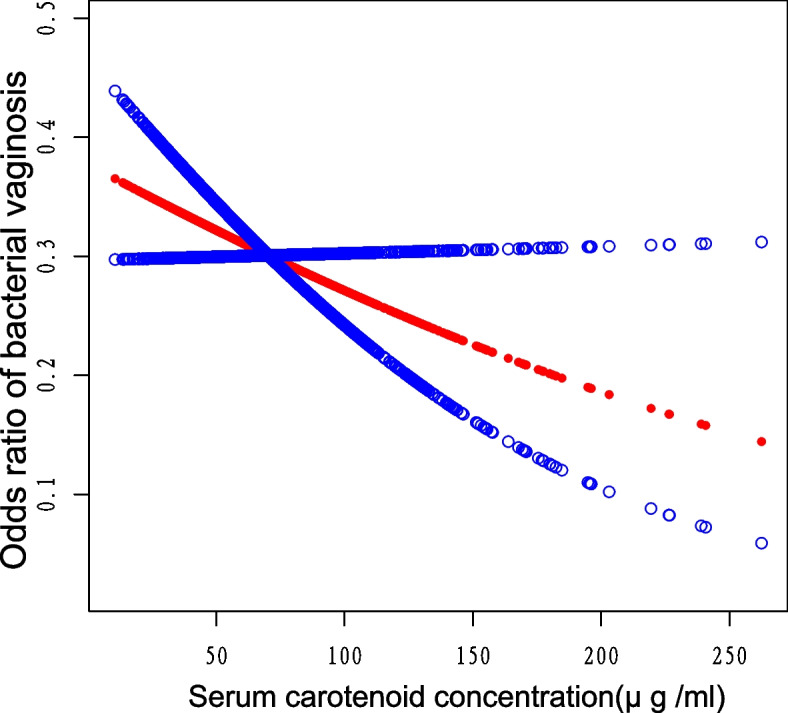
Correlation of total serum carotenoids with BV. The central red dots represent serum carotenoid concentrations, with each point contributing to a continuous fitted curve. The region between the two blue dashed lines corresponds to the 95% confidence interval. The X-axis is serum carotenoid levels (continuous variable), and the Y-axis is odds ratios (ORs). ORs were computed from Model II in a multivariate logistic regression analysis

Table 2 displays the correlations between quartile total serum carotenoids and BV among the three models. Compared with the remaining three groups, the prevalence of BV was lowest in the group with the highest total serum carotenoid content (Q4) [Unadjusted model: OR = 0.38 (0.27, 0.54), *P* < 0.0001, Model I: OR = 0.50 (0.33, 0.74), *P* = 0.0005, Model II: OR = 0.63 (0.41, 0.96), *P* = 0.0304]. We visualized the connection between serum carotenoid levels and BV by creating a smooth curve fitting and assessing the linear relationship between them. As shown in Fig. 2, the relationship between serum carotenoids and BV was negative linear and statistically significant (*P* = 0.0362). The smooth curve fitting plot indicated a decrease in the incidence of BV with increasing serum total carotenoid concentrations. In summary, there was an inverse association between total serum carotenoids and the occurrence of BV.

**Table 2:** Association of total serum carotenoids with BV

	Unadjusted model		Model I		Model II	
	OR (95% CI)	*P* value	OR (95% CI)	*P* value	OR (95% CI)	*P* value
Serum carotenoids	0.99 (0.98, 0.99)	< 0.0001	0.99 (0.99, 1.00)	0.0019	0.99 (0.99, 1.00)	0.0699
Serum carotenoids quartile						
Q1 (3.14–47.69 μg /ml)	reference		reference		reference	
Q2 (47.77–64.39 μg /ml)	0.57 (0.41, 0.80)	0.0011	0.63 (0.44, 0.91)	0.0129	0.67 (0.46, 0.96)	0.0313
Q3 (64.40–85.80 μg /ml)	0.61 (0.44, 0.85)	0.0035	0.72 (0.50, 1.03)	0.0751	0.77 (0.53, 1.13)	0.1790
Q4 (85.96–331.7 μg /ml)	0.38 (0.27, 0.54)	< 0.0001	0.50 (0.33, 0.74)	0.0005	0.63 (0.41, 0.96)	0.0304

Unadjusted model: no covariates were adjusted

Model I: age, race, education status, BMI, marital status, PIR, and physical activity (Moderate and vigorous activity) were adjusted

Model II: age, race, education status, BMI, marital status, PIR, physical activity (Moderate and vigorous activity), C-reactive protein, serum vitamin A, serum vitamin E, serum calcium, high cholesterol level, sexual intercourse, birth control pills, smoking status, and alcohol consumption, were adjusted

### The association of prime components of serum carotenoids with BV

Table 3 presents the results of multiple logistic regression for the prime components of serum carotenoids. When comparing quartiles Q3 and Q4, α-carotene and β-cryptoxanthin showed significant negative correlations with BV (OR < 1, *p* < 0.05) in all three models. Lutein and zeaxanthin showed a significant negative correlation only at Q4 in the unadjusted model and in model I. However, in model II, the relationship between β-carotene and BV was not strong [Q2: OR = 0.85 (0.64, 1.12), *p* = 0.2512; Q3: OR = 0.77 (0.57, 1.02), *p* = 0.0726; Q4: OR = 0.78 (0.57, 1.07), *p* = 0.1257]. The correlations between lycopene and BV were not statistically significant (p < 0.05) in all models.

**Table 3:** Association between prime components of serum carotenoids with BV

	Unadjusted model		Model I		Model II	
	OR (95% CI)	*P* value	OR (95% CI)	*P* value	OR (95% CI)	*P* value
α-Carotene	0.87 (0.82, 0.91)	< 0.0001	0.93 (0.88, 0.98)	0.0099	0.96 (0.91, 1.02)	0.2122
α-Carotene quartile						
Q1(0.21–1.37 μ g /ml)	reference		reference		reference	
Q2 (1.38–2.59 μ g /ml)	0.46 (0.33, 0.65)	< 0.0001	0.57 (0.40, 0.82)	0.0025	0.63 (0.43, 0.91)	0.0132
Q3(2.60–5.12 μ g /ml)	0.36 (0.26, 0.50)	< 0.0001	0.49 (0.34, 0.72)	0.0002	0.57 (0.38, 0.85)	0.0052
Q4(5.14–69.2 μ g /ml)	0.31 (0.22, 0.44)	< 0.0001	0.50 (0.34, 0.75)	0.0007	0.64 (0.42, 0.99)	0.0433
β-Carotene	0.97 (0.96, 0.98)	< 0.0001	0.98 (0.97, 0.99)	0.0171	0.99 (0.98, 1.00)	0.3357
β-Carotene quartile						
Q1 (0.79–7.58 μ g /ml)	reference		reference		reference	
Q2 (7.59–12.74 μ g/ml)	0.55 (0.39, 0.76)	0.0004	0.62 (0.44, 0.89)	0.0101	0.69 (0.48, 1.00)	0.0495
Q3 (12.76–22.44 μ g/ml)	0.57 (0.41, 0.80)	0.0010	0.70 (0.48, 1.01)	0.0558	0.83 (0.57, 1.21)	0.3336
Q4(22.46–193 μ g /ml)	0.37 (0.26, 0.53)	< 0.0001	0.55 (0.37, 0.82)	0.0031	0.72 (0.47, 1.11)	0.1375
β-Cryptoxanthin	0.96 (0.93, 0.98)	0.0005	0.96 (0.93, 0.98)	0.0023	0.97 (0.94, 1.00)	0.0535
β-Cryptoxanthin quartile						
Q1 (0.14–5.23 μ g /ml)	reference		reference		reference	
Q2 (5.25–8.07 μ g /ml)	0.83 (0.60, 1.15)	0.2630	0.74 (0.51, 1.06)	0.1035	0.77 (0.53, 1.12)	0.1687
Q3(8.10–13.47 μ g /ml)	0.48 (0.34, 0.68)	< 0.0001	0.49 (0.33, 0.72)	0.0003	0.56 (0.37, 0.83)	0.0039
Q4 (13.49–99.10 μ g /ml)	0.56 (0.40, 0.79)	0.0009	0.52 (0.35, 0.78)	0.0018	0.63 (0.41, 0.98)	0.0393
Lycopene	0.99 (0.97, 1.00)	0.1225	0.99 (0.97, 1.01)	0.2132	0.99 (0.98, 1.01)	0.3707
Lycopene quartile						
Q1 (0.68–15.9 μ g /ml)	reference		reference		reference	
Q2 (15.96–21.59 μ g /ml)	0.84 (0.60, 1.17)	0.2999	0.89 (0.62, 1.29)	0.5424	0.95 (0.65, 1.38)	0.7841
Q3 (21.60–28.46 μ g /ml)	0.93 (0.67, 1.31)	0.6887	1.00 (0.70, 1.43)	0.9987	1.02 (0.70, 1.47)	0.9245
Q4 (28.50–81.47 μ g /ml)	0.73 (0.52, 1.03)	0.0764	0.77 (0.53, 1.11)	0.1561	0.82 (0.56, 1.21)	0.3209
Lutein / Zeaxanthin	0.96 (0.94, 0.98)	< 0.0001	0.97 (0.94, 0.99)	0.0055	0.97 (0.95, 1.00)	0.0512
Lutein / Zeaxanthin quartile						
Q1 (0.14–10.27 μ g /ml)	reference		reference		reference	
Q2 (10.28–13.71 μ g /ml)	0.86 (0.62, 1.20)	0.3822	0.86 (0.60, 1.23)	0.4183	0.91 (0.63, 1.32)	0.6261
Q3 (13.72–18.47 μ g /ml)	0.78 (0.55, 1.09)	0.1386	0.79 (0.55, 1.15)	0.2227	0.86 (0.59, 1.26)	0.4437
Q4 (18.50–69.30 μ g /ml)	0.50 (0.35, 0.71)	< 0.0001	0.58 (0.39, 0.86)	0.0063	0.67 (0.44, 1.01)	0.0562

Unadjusted model: no covariates were adjusted

Model I: age, race, education status, BMI, marital status, PIR, and physical activity (Moderate and vigorous activity) were adjusted

Model II: age, race, education status, BMI, marital status, PIR, physical activity (Moderate and vigorous activity), C-reactive protein, serum vitamin A, serum vitamin E, serum calcium, high cholesterol level, sexual intercourse, birth control pills, smoking status, and alcohol consumption, were adjusted

**Fig. 3:**
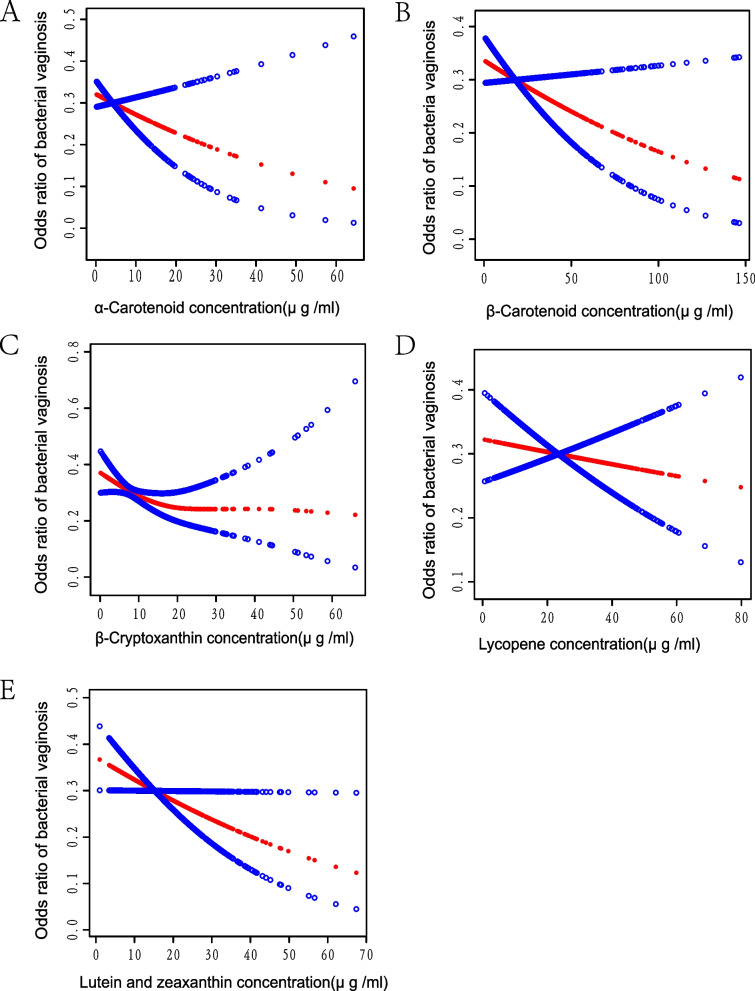
Correlation between prime components of serum carotenoids and BV. Respectively, Fig. 3 A, B, C, D, and E represent the correlation between α-carotene, β-carotene, β-cryptoxanthin, lycopene, lutein/zeaxanthin, and BV. The central red dots represent serum carotenoid concentrations, with each point contributing to a continuous fitted curve. The region between the two blue dashed lines corresponds to the 95% confidence interval. The X-axis is serum carotenoid levels (continuous variable), and the Y-axis is odds ratios (ORs). ORs were computed from Model II in a multivariate logistic regression analysis

As shown in Fig. 3, we employed smooth curve fitting to depict the association between the primary components of serum carotenoids and BV. The six smoothed curve fittings demonstrated that serum carotenoids were negatively associated with the occurrence of BV. We did not identify any potential non-linear relationships between the six primary carotenoids and the incidence of BV. Noteworthy, only the linear relationship between lutein/zeaxanthin and BV was significant (*P* = 0.0458).

### Stratified analysis between total serum carotenoids and BV

As shown in Table 4, demographically relevant covariates such as age, race, BMI, birth control pills, smoking status, and alcohol consumption were stratified separately. In general, the trend of negative correlation of OR across subgroups was relatively stable (OR < 1). Serum total carotenoids were significantly (*P* < 0.05) negatively correlated with BV at Q2 in females aged between 40 and 49 years, non-Hispanic whites, overweight individuals (24.9 < BMI < 30), smokers, and alcoholics. Furthermore, in women aged 26–33 years, non-Hispanic blacks, and contraceptive pill users, serum total carotenoids exhibited significant negative correlations with BV in Q4 (P < 0.05). For underweight women, a stratified statistical analysis based on BMI was not feasible due to an insufficient sample size.

**Table 4:** Stratified analysis between total serum carotenoids and BV

Stratified variable	N	Serum carotenoids concentration			
		Q1 (3.14–47.69 μg /ml)	Q2 (47.77–64.39 μg /ml)	Q3 (64.40–85.80 μg /ml)	Q4(85.96–331.7 μg /ml)
Age (year)					
20–25	283	1.0	0.79 (0.36, 1.75) 0.5596	1.02 (0.46, 2.26) 0.9692	2.30 (0.83, 6.36) 0.1074
26–33	341	1.0	0.78 (0.34, 1.76) 0.5451	0.78 (0.34, 1.77) 0.5534	0.32 (0.12, 0.86) 0.0234
34–40	277	1.0	0.76 (0.32, 1.78) 0.5262	0.68 (0.28, 1.63) 0.3826	0.55 (0.21, 1.45) 0.2304
41–49	351	1.0	0.40 (0.19, 0.84) 0.0164	0.59 (0.26, 1.34) 0.2063	0.44 (0.19, 1.01) 0.0528
Race					
Non-Hispanic White	678	1.0	0.51 (0.30, 0.88) 0.0145	0.80 (0.46, 1.41) 0.4376	0.60 (0.31, 1.15) 0.1257
Non-Hispanic Black	227	1.0	1.28 (0.55, 2.97) 0.5622	0.77 (0.33, 1.78) 0.5392	0.23 (0.07, 0.75) 0.0152
Other Race	347	1.0	0.81 (0.36, 1.82) 0.6131	1.14 (0.52, 2.50) 0.7401	1.33 (0.59, 3.03) 0.4903
BMI (kg/m2)					
Healthy Weight	471	1.0	0.86 (0.43, 1.74) 0.6779	0.89 (0.44, 1.79) 0.7379	0.60 (0.28, 1.29) 0.1906
Overweight	349	1.0	0.29 (0.13, 0.66) 0.0032	0.49 (0.22, 1.06) 0.0713	0.50 (0.22, 1.15) 0.1044
Obesity	398	1.0	0.97 (0.54, 1.74) 0.9217	1.20 (0.62, 2.31) 0.5851	0.73 (0.32, 1.66) 0.4521
Birth control pills					
Yes	1018	1.0	0.68 (0.46, 1.02) 0.0649	0.73 (0.48, 1.12) 0.1535	0.52 (0.32, 0.84) 0.0073
No	234	1.0	0.58 (0.22, 1.55) 0.2755	0.74 (0.30, 1.83) 0.5129	0.98 (0.36, 2.67) 0.9738
Smoking status					
Smokers	579	1.0	0.52 (0.32, 0.87) 0.0121	0.67 (0.39, 1.14) 0.1368	0.54 (0.28, 1.04) 0.0635
Non-smokers	673	1.0	0.98 (0.54, 1.76) 0.9457	0.99 (0.55, 1.78) 0.9630	0.77 (0.42, 1.44) 0.4197
Alcohol consumption					
Nondrinker	449	1.0	0.82 (0.40, 1.69) 0.5914	0.80 (0.38, 1.68) 0.5592	0.55 (0.24, 1.24) 0.1497
Moderate alcohol use	401	1.0	0.56 (0.28, 1.12) 0.1013	0.69 (0.33, 1.43) 0.3199	0.75 (0.33, 1.67) 0.4767
Alcoholism	402	1.0	0.52 (0.28, 0.94) 0.0307	0.75 (0.40, 1.42) 0.3802	0.48 (0.22, 1.01) 0.0539

Model II: age, race, education status, BMI, marital status, PIR, physical activity (Moderate and vigorous activity), C-reactive protein, serum vitamin A, serum vitamin E, serum calcium, high cholesterol level, sexual intercourse, birth control pills, smoking status, and alcohol consumption, were adjusted

In the stratified analyses for a given covariate, that specific covariate was not included in the adjustment model. The stratified analysis exclusively employed Model II

### Sensitivity analysis

The study used multiple interpolations to populate the missing values of covariates for sensitivity analysis. The direction of the results of the sensitivity analyses (Supplementary Table 1) was generally consistent with the formal results, except that β-carotene became significant in Model 2.

## Discussion

As far as we know, this finding represents the first cross-sectional investigation into the association between prime serum carotenoid concentrations and patients diagnosed with BV. The results of this study demonstrated that heightened serum carotenoid concentrations were associated with a diminished prevalence of BV. Specifically, serum α-carotene and β-cryptoxanthin concentrations exhibited significant correlations with reduced prevalence of BV. Conversely, lycopene did not demonstrate a significant association with the prevalence of BV. The reliability of the results was confirmed by performing different stratification and sensitivity analyses. Therefore, improving serum carotenoid status in women may provide a biological rationale for the clinical prevention of bacterial vaginosis infection and prevention of recurrence.

Carotenoids are abundantly present in various vegetables and fruits and constitute a significant category of micronutrients [42]. Recent research has demonstrated that adhering to a plant-based diet or increasing the consumption of antioxidant-rich vegetables is linked to a reduced incidence of BV [43–46]. Research conducted by Tohill et al. revealed that specific micronutrient deficiencies were linked to an elevated occurrence of BV, encompassing deficiencies in vitamin A, β-carotene, vitamin E, and vitamin C [33]. Furthermore, a randomized controlled trial indicated that increased consumption of β-carotene and vitamin A was associated with a reduced prevalence of BV [47]. However, past studies have focused primarily on the correlation between β-carotene and BV, ignoring other carotenoids. Our results indicated that the risk of developing BV decreases with an increase in serum carotenoids, especially in α-carotene and β-cryptoxanthin, suggesting that some serum carotenoids may influence the occurrence of BV. However, the precise mechanisms underlying the impact of serum carotenoids on BV remain unclear.

Noteworthy, in the results of the fully adjusted model, the negative correlation between β-carotene and BV was not significant but became significant in the sensitivity analyses. Such discrepancy was also reported in some previous studies. For example, a prospective study suggests that the intake of specific nutrients is unrelated to BV, including β-carotene [32]. Additionally, a case-control study indicates an association between α-carotene and cervical abnormalities in women, while other types of carotenoids do not show such a connection [48]. Exploring the exact link between β-carotene and BV may require prospective cohort studies with larger sample sizes or mechanistic studies.

Several potential biological mechanisms may elucidate the significant role of carotenoids in preventing the onset of BV. BV often coincides with an imbalance in vaginal flora and an increase in opportunistic pathogenic bacteria, which can result in an accumulation of reactive oxygen species in the vaginal environment [19]. Carotenoids, as potent antioxidants, can effectively mitigate the buildup of reactive oxygen species and sustain flora diversity [49]. Moreover, research has found that a woman’s mucosal immunity plays a pivotal role in the prevention of BV, which involves vaginal epithelial cells, local lymphoid tissue, and some functional enzymes [50]. Regarding the maintenance of vaginal epithelial cells, adequate carotenoids and vitamin A were found to be essential [51]. Simultaneously, innate and adaptive immunological protection is indispensable for the mucosal surfaces of the female genital tract [52]. Several carotenoids have been demonstrated to possess the capacity to stimulate the proliferation and differentiation of various lymphocytes, thereby strengthening the body’s immune system [53]. In particular, carotenoids, including α-carotene, β-carotene, and β-cryptoxanthin enhance the function of natural killer cells, neutrophils, and other innate immune cells [18, 54, 55].

Several limitations of this study warrant acknowledgment. Firstly, the utilization of a cross-sectional design precluded the establishment of a definitive causal relationship between bacterial vaginosis and serum carotenoids. A longitudinal study would be more suitable for elucidating the causal association between these variables. Secondly, it is plausible that intricate additive effects and biological interactions exist among various nutrients and non-nutrient factors, but the scope of this study does not cover these aspects. Additionally, behavioral habits, including the frequency of sexual activity and the frequency of partner changes, may exert an influence on the outcome [56]. Although we incorporated multiple covariates for adjustment, the potential for residual confounding remains. Lastly, since only single baseline measurements of serum carotenoid concentrations were employed, it was not feasible to evaluate the time-varying correlation.

## Conclusion

In a nutshell, the cross-sectional study showed that serum carotenoids correlated negatively with bacterial vaginosis. Consuming more carotenoid-rich fruits and vegetables or taking carotenoid supplements may prevent the onset and recurrence of bacterial vaginosis. More specific mechanisms of influence need further experimental verification.

### Supplementary Information

**Additional file 1.**

## Data Availability

The dataset that provides the necessary information and evidence to support the conclusions is accessible in the NHANES repository, https://www.cdc.gov/nchs/nhanes/index.htm.
