# Supplementary material for: Association between serum carotenoids and bacterial vaginosis infection among American women
Source: BMC Infect Dis. 2024 Jan 2;24:20. doi: 10.1186/s12879-023-08908-3 (PMC10762845; doi:10.1186/s12879-023-08908-3)
Supplement: Supplementary file 1 — Additional file 1. [file 12879_2023_8908_MOESM1_ESM.docx]

Supplementary Table 1 Sensitivity analysis of main components of Serum Carotenoids in relation to BV

|  | Unadjusted model | | Model I | | Model II | |
| --- | --- | --- | --- | --- | --- | --- |
|  | OR (95% CI) | P value | OR (95% CI) | P value | OR (95% CI) | P value |
| Serum carotenoids | 0.99 (0.98, 0.99) | <0.0001 | 0.99 (0.99, 1.00) | <0.0001 | 0.99 (0.99, 1.00) | 0.0004 |
| Serum carotenoids quartile |  | | | | | |
| Q1 （3.14-46.49μg /ml) | reference | | reference | | reference | |
| Q2 （46.54-62.72μg /ml) | 0.67 (0.54, 0.85) | 0.0007 | 0.71(0.56,0.90) | 0.0054 | 0.73 (0.57, 0.93) | 0.0108 |
| Q3 （62.73-84.69μg /ml) | 0.69 (0.55, 0.86) | 0.0013 | 0.77 (0.61, 0.99) | 0.0414 | 0.83 (0.65, 1.07) | 0.1446 |
| Q4 （84.70-331.7μg /ml) | 0.43 (0.34, 0.55) | <0.0001 | 0.50 (0.39, 0.65) | <0.0001 | 0.57 (0.43, 0.75) | <0.0001 |
|  | | | | | | |
| α-Carotene | 0.87 (0.84, 0.90) | <0.0001 | 0.91 (0.88, 0.95) | <0.0001 | 0.95 (0.90, 0.97) | 0.0004 |
| α-Carotene quartile |  | | | | | |
| Q1（0.21-1.27 μ g /ml) | reference | | reference | | reference | |
| Q2 （1.28-2.39 μ g /ml) | 0.61 (0.48, 0.76) | <0.0001 | 0.73 (0.57, 0.92) | 0.0093 | 0.75 (0.59, 0.96) | 0.0221 |
| Q3（2.40-4.85 μ g /ml) | 0.50 (0.40, 0.63) | <0.0001 | 0.65 (0.51, 0.84) | 0.0009 | 0.71 (0.55, 0.92) | 0.0098 |
| Q4（4.86-69.2 μ g /ml) | 0.35 (0.27, 0.44) | <0.0001 | 0.49 (0.37, 0.64) | <0.0001 | 0.56 (0.42, 0.75) | <0.0001 |
|  | | | | | | |
| β-Carotene | 0.97 (0.96, 0.98) | <0.0001 | 0.98 (0.97, 0.99) | 0.0001 | 0.99 (0.98, 1.00) | 0.0106 |
| β-Carotene quartile |  | | | | | |
| Q1 （0.79-7.28 μ g /ml) | reference | | reference | | reference | |
| Q2 （7.29-11.86 μ g/ml) | 0.63 (0.50, 0.79) | <0.0001 | 0.74 (0.58, 0.94) | 0.0123 | 0.77 (0.61, 0.99) | 0.0378 |
| Q3 （11.87-21.36 μ g/ml) | 0.56 (0.44, 0.70) | <0.0001 | 0.67 (0.53, 0.86) | 0.0017 | 0.74 (0.57, 0.95) | 0.0200 |
| Q4（21.40-210 μ g /ml) | 0.42 (0.33, 0.53) | <0.0001 | 0.57 (0.44, 0.74) | <0.0001 | 0.66 (0.50, 0.87) | 0.0036 |
|  | | | | | | |
| β-Cryptoxanthin | 0.96 (0.93, 0.98) | <0.0001 | 0.96 (0.93, 0.98) | <0.0001 | 0.97 (0.94, 1.00) | 0.0002 |
| β-Cryptoxanthin quartile |  | | | | | |
| Q1 （0.14-5.05 μ g /ml) | reference | | reference | | reference | |
| Q2 （5.06-7.68 μ g /ml) | 0.81 (0.65, 1.02) | 0.0705 | 0.75 (0.59, 0.96) | 0.0207 | 0.78 (0.61, 1.00) | 0.0462 |
| Q3（7.70-12.74 μ g /ml) | 0.73 (0.58, 0.92) | 0.0066 | 0.70 (0.54, 0.90) | 0.0053 | 0.76 (0.59, 0.99) | 0.0387 |
| Q4 （12.79-99.10 μ g /ml) | 0.52 (0.41, 0.66) | <0.0001 | 0.48 (0.36, 0.63) | <0.0001 | 0.55 (0.41, 0.74) | <0.0001 |
|  | | | | | | |
| Lycopene | 0.99 (0.99, 1.00) | 0.0419 | 0.99 (0.98, 1.01) | 0.1127 | 0.99 (0.98, 1.00) | 0.1458 |
| Lycopene quartile |  | | | | | |
| Q1 （0.68-15.9 μ g /ml) | reference | | reference | | reference | |
| Q2 （15.96-21.59 μ g /ml) | 0.87 (0.69, 1.09) | 0.2221 | 0.93 (0.73, 1.19) | 0.5424 | 0.95 (0.74, 1.22) | 0.6929 |
| Q3 （21.60-28.46 μ g /ml) | 0.97 (0.77, 1.22) | 0.7951 | 1.06 (0.84, 1.36) | 0.9987 | 1.07 (0.84, 1.37) | 0.5813 |
| Q4 （28.50-81.47 μ g /ml) | 0.76 (0.60, 0.96) | 0.0191 | 0.79 (0.62, 1.02) | 0.1561 | 0.80 (0.62, 1.04) | 0.0920 |
|  | | | | | | |
| Lutein / Zeaxanthin | 0.96 (0.95, 0.98) | <0.0001 | 0.97 (0.95, 0.98) | <0.0001 | 0.97 (0.95, 0.99) | 0.0011 |
| Lutein / Zeaxanthin quartile |  | | | | | |
| Q1 （0.14-10.27 μ g /ml) | reference | | reference | | reference | |
| Q2 （10.28-13.71 μ g /ml) | 0.97 (0.77, 1.21) | 0.7643 | 0.88 (0.69, 1.13) | 0.3174 | 0.92 (0.72, 1.18) | 0.5159 |
| Q3 （13.72-18.47 μ g /ml) | 0.82 (0.65, 1.03) | 0.0857 | 0.77 (0.60, 0.99) | 0.0432 | 0.82 (0.64, 1.06) | 0.1227 |
| Q4 （18.50-69.30 μ g /ml) | 0.58 (0.46, 0.74) | <0.0001 | 0.59 (0.45, 0.77) | <0.0001 | 0.65 (0.49, 0.85) | 0.0019 |

Unadjusted model: no covariates were adjusted.

Model I: age, race, education status, BMI, marital status, PIR, and physical activity (Moderate and vigorous activity) were adjusted.

Model II: age, race, education status, BMI, marital status, PIR, physical activity (Moderate and vigorous activity), C-reactive protein, serum vitamin A, serum vitamin E, serum calcium, high cholesterol level, sexual intercourse, birth control pills, smoking status, and alcohol consumption, were adjusted.
